# Analysis of the Papillomavirus E2 and Bromodomain Protein Brd4 Interaction Using Bimolecular Fluorescence Complementation

**DOI:** 10.1371/journal.pone.0077994

**Published:** 2013-10-25

**Authors:** Christine M. Helfer, Ranran Wang, Jianxin You

**Affiliations:** Department of Microbiology, University of Pennsylvania Perelman School of Medicine, Philadelphia, Pennsylvania, United States of America; International Centre for Genetic Engineering and Biotechnology, Italy

## Abstract

The human papillomavirus (HPV) vaccines effectively protect against new infections of up to four HPV subtypes. However, these vaccines are not protective against many other clinically relevant HPV subtypes and are ineffective at treating established HPV infections. There is therefore a significant need for antiviral treatments for persistent HPV infections. A promising anti-HPV drug target is the interaction between the HPV E2 protein and cellular bromodomain-containing protein 4 (Brd4) since this protein complex mediates several processes important for the viral life cycle including viral genome maintenance, replication, and transcription. Using bimolecular fluorescence complementation (BiFC) technology, we demonstrate the E2 and Brd4 interaction on both interphase chromatin and mitotic chromosomes throughout mitosis. The E2-Brd4 BiFC was significantly diminished by mutating the Brd4 binding sites in E2 or by a dominant negative inhibitor of the E2-Brd4 interaction, demonstrating the potential of BiFC for identifying inhibitors of this important virus-host interaction. Importantly, when Brd4 was released from chromatin using the bromodomain inhibitor JQ1(+), the E2-Brd4 interacting complex relocated into foci that no longer associate with mitotic chromosomes, pointing to JQ1(+) as a promising antiviral inhibitor of HPV genome maintenance during HPV persistent infection.

## Introduction

Human papillomavirus (HPV) is one of the most common sexually transmitted pathogens in the world. Over 150 HPV subtypes exist, with low-risk subtypes causing anogenital warts, while high-risk subtypes are associated with cervical and anal cancers as well as head and neck cancers [1]. Cervical cancer is one of the leading causes of cancer-related death in women, killing roughly 288,00 women every year with HPV subtypes 16 and 18 responsible for over 70% of cervical cancer cases [2–4].

The papillomavirus (PV) life cycle is intimately linked to the differentiation program of the infected keratinocyte. Infection begins in the basal epithelial cells where viral genomes are maintained as extra-chromosomal circular genomes called episomes that replicate along with the cellular DNA [5]. Subsequent differentiation of the infected epithelial cell triggers PV genome amplification, expression of viral capsid proteins, and assembly of infectious virions [5].

 There are now two commercially available prophylactic HPV virus-like particle vaccines. These vaccines protect against HPV types 16 and 18 (Cervarix) and HPV types 6, 11, 16, and 18 (Gardasil). Both vaccines are highly effective against HPV 16/18, and Gardasil has shown greater than 98% efficacy against HPV 6/11-associated genital warts [6–8]. Although these vaccines will likely reduce the incidence of HPV-associated diseases in the long-term, they do not protect against the other high-risk HPV types and are ineffective at treating established infections [9,10]. Alternative approaches are therefore needed for curing ongoing HPV infections. This is particularly important because high-risk HPVs need to persistently infect host cells for years or even decades in order to accumulate substantial cytogenetic changes for developing invasive tumors.

Despite their potential benefits, there are currently no virus-specific antiviral therapies for HPV infection. Since the E1 helicase protein is the only enzyme encoded by papillomaviruses, it is a promising target for drug design. Small molecule inhibitors against E1 ATPase activity were identified but were unfortunately found to be inactive in cellular assays [11,12]. The highly conserved papillomavirus E2 protein interacts with E1 and is required for viral genome replication, making this protein another promising candidate for drug targeting. Indeed, White et al. identified small molecule inhibitors of the E2-E1 interaction for HPV 6 and 11, demonstrating the feasibility of developing small molecule inhibitors for virus-mediated protein-protein interactions [13]. 

The PV E2 protein contains an N-terminal transactivation domain connected by a flexible hinge to the C-terminal DNA binding domain [14]. Besides initiating PV genome replication via recruiting E1 to the viral origin, E2 also regulates viral gene transcription, which is largely mediated by the interaction between the N-terminal transactivation domain and cellular proteins [15]. E2 transactivates the PV early promoter by recruiting transcription regulatory factors including CBP, p/CAF, BRCA1, Brm, and bromodomain-containing protein 4 (Brd4) [16–21]. Conversely, E2 can also repress viral transcription by blocking transcription factor binding to the early promoter and recruiting chromatin modifying factors [22–25]. PVs establish persistent latent infection in basal epithelial cells where the virus stably maintains a low copy number of viral genomes. E2 also functions in viral genome maintenance during persistent infection by tethering PV genomes to mitotic chromosomes to ensure their faithful partitioning into daughter cells during cell divisions [26–30].

An important E2 interacting partner is Brd4 [31]. Brd4 is a BET family member that binds acetylated histones with two conserved bromodomains and remains associated with chromosomes during mitosis [32]. The C-terminal domain (CTD) of Brd4 has been shown to interact with the E2 transactivation domain of most PVs including the HPV subtypes [20]. Brd4 and bovine papillomavirus 1 (BPV1) E2 interact on mitotic chromosomes to mediate BPV1 viral genome maintenance [31,33–35]. Additional evidence suggests that Brd4 is necessary for HPV16 and HPV31 episome tethering to mitotic chromosomes [36]. However, whether these HPV E2s also bind Brd4 on mitotic chromosomes have not been clearly demonstrated. Brd4 is also an important cofactor for E2 transcription activation [20,21,25,37]. A luciferase-based protein complementation assay has been described to show that Brd4 binding can increase the E2 protein stability [38]. In addition, a recent report from our laboratory revealed that Brd4 is an essential component of the HPV genome replication complex [39].

Since E2 in complex with Brd4 controls multiple important HPV functions, it has been proposed that antiviral inhibitors targeting this interaction would likely abrogate the HPV life cycle, resulting in clearance of the infection [11]. Indeed it has already been demonstrated that blocking the E2-Brd4 interaction with either Brd4 binding-deficient E2 mutants or expression of the Brd4 CTD impairs viral transcription activation and HPV genome replication [20,21,39]. Furthermore, abrogation of the E2-Brd4 interaction also abolishes tethering of both HPV16 and HPV31 viral episomes to mitotic chromosomes, which could eventually result in clearance of the viral genomes as cells divide [36].

Screening for inhibitors of protein-protein interactions requires a robust and efficient assay. In this study, we demonstrate the usefulness of bimolecular fluorescence complementation (BiFC) as an effective tool for identifying inhibitors of protein-protein interactions. We focused on the E2-Brd4 interaction since it is a promising target for HPV antiviral therapy. With BiFC technology, we visualized physiological levels of E2 and Brd4 interacting in live cells at all cell cycle stages. Specificity of this interaction was confirmed using E2 mutants that abolish Brd4 binding. We also demonstrate the tethering of HPV16 E2 to mitotic chromosomes through Brd4. Furthermore, the utility of BiFC for drug screening is demonstrated using Brd4 CTD as an E2-Brd4 inhibitor, which effectively abolishes the E2-Brd4 BiFC signal. Finally, we show that the bromodomain inhibitor, JQ1(+), releases E2-Brd4 BiFC from mitotic chromosomes, identifying this drug as a potential agent to interrupt and clear HPV persistent infection.

## Materials and Methods

### Recombinant plasmid construction

The plasmid encoding the Xpress-tagged CTD (pcDNA4C-NLS-CTD) has been described previously [31,40]. The cloning strategy for BiFC constructs has been described previously [41]. To construct Brd4 or E2 fusions with VN (encoding Venus N for Venus aa1-155) or VC (encoding Venus C for Venus aa156-238), a short linker sequence (GGSGG) was introduced in the C-terminal end of VN/VC fragments by PCR, and the amplified DNA fragments were cloned into the pOZN vector at the XhoI site. Brd4, E2TA, E2TR or 16E2 DNA fragments excised from their pOZN constructs using XhoI and NotI digestion were ligated into pOZN-VN-short linker and pOZN-VC-short linker to generate in-frame fusions of these molecules with either Venus N or Venus C. The pOZN-VN constructs used in BiFC experiments as the negative control have been described previously [41]. The R37A/I73A 16E2 mutant was generated by site-directed mutagenesis of the pOZN-VC-16E2 construct using the QuikChange Site-Directed Mutagenesis Kit (Stratagene). The pcDNA4C-NLS-LacI plasmid was generated by cloning a nuclear localization sequence (NLS) into the pcDNA4C vector using a BamHI site and the PCR-amplified LacI cDNA fragment into the pcDNA4C vector using BamHI and EcoRI sites. The pEFHPV-16W12E plasmid was a gift from Paul Lambert [42]. All plasmid constructs were verified by DNA sequencing.

### Cell culture and transfection

C33A cells were purchased from ATCC and maintained as monolayers in high glucose Dulbecco’s modified Eagle’s medium (DMEM, GIBCO) containing 10% fetal bovine serum (FBS, Hyclone). FuGENE 6 (Roche) and Lipofectamine 2000 (Invitrogen) transfection reagents were used for transient transfection, following the manufacturer’s instructions. 

### Immunofluorescent staining

Cells cultured on cover slips were fixed with 3% paraformaldehyde in PBS for 20 min. Immunofluorescent staining was performed as previously described [40,43]. The following primary antibodies were used: anti-Xpress (Invitrogen), anti-HA (Santa Cruz), and anti-FLAG (Sigma). Secondary antibodies used were: Alexa Fluor 594 goat anti-rabbit IgG (Invitrogen), Alexa Fluor 594 goat anti-mouse IgG (Invitrogen) and Alexa Fluor 350 goat anti-mouse IgG (Invitrogen). The Brd4 inhibitor, JQ1(+), and its enantiomer, JQ1(-), were dissolved in dimethyl sulfoxide (DMSO) as 1000X stocks as previously described [44].

### Microscopy and image analysis

All immunofluorescence images were collected using an inverted fluorescence microscope (Olympus, IX81) equipped with an UPlanSApo 40×/0.95 NA lens (Olympus), an UPlanSApo 100×/1.4 oil immersion lens (Olympus) and a high-resolution charge-coupled device camera (QImaging, FAST1394) at room temperature. Photos were taken using either a 40× or 100× lens with immersion oil type-F (Olympus). Image data were analyzed and presented using SlideBook 5.0 software (Intelligent Imaging Innovations, Inc.). The scale bars were added using ImageJ software.

 For Figure S1, immunofluorescent images were analyzed using ImageJ software. The “Adjust Threshold” function of the ImageJ software was used to identify DAPI-stained nuclei. The average BiFC signal intensity divided by nucleus area was measured using the “Analyze Particles” function of the software. The BiFC signal intensity of 50 cells transfected with vector and 50 cells transfected with HPV16 genome was measured and divided by the nucleus area to get the values plotted in Figure S1. This experiment was repeated 2 times with similar results.

### Western blot analysis

For all Western blots, C33A cells were collected at 48 h post-transfection and washed once with PBS. The cell pellets were lysed in buffer A (10 mM HEPES, pH 7.9, 10 mM KCl, 0.1 mM EDTA, 0.1 mM EGTA, and 1 mM dithiothreitol (DTT), supplemented with protease inhibitors (Roche)). The cells were incubated on ice for 10 min, and NP-40 was added to a final concentration of 0.6%. After vortexing and centrifugation at 5,000 rpm for 5 min, the nuclear pellet was resuspended in ice-cold buffer B (20 mM HEPES, pH 7.9, 0.4 M NaCl, 0.1 mM EDTA, 0.1 mM EGTA, and 1 mM DTT, supplemented with protease inhibitors) and incubated on ice for 15 min with vortexing. The nuclear proteins were isolated by centrifugation at 14,000 rpm for 5 min. The samples were then resolved on an SDS-PAGE gel, transferred onto PVDF membrane, and immunoblotted with specific antibodies as indicated in the figure legends. Antibodies employed in the Western blot analysis include: anti-Brd4C (recognizing Brd4 aa1313-1362), anti-Xpress (Invitrogen), anti-Actin (Chemicon), and anti-HA-HRP (Roche). Western blots were developed using ECL solution (PerkinElmer) and images were captured using a Fuji imaging system.

## Results

### Demonstration of the E2 and Brd4 interaction using BiFC

In this study we employed BiFC technology to examine the E2-Brd4 interaction in live cells. BiFC involves fusing two proteins individually to complementary fragments of a fluorescent reporter protein and expressing them together in live cells. Interaction of these proteins brings the reporter protein fragments within close proximity, allowing the fluorescent protein to reform its native three-dimensional structure and emit fluorescent signal [45]. Using this method, protein interactions can be identified and located within the cell through visualization of the intensity and distribution of fluorescence in these cells. BiFC technology has been previously used to examine Brd4 intermolecular interactions as well as Brd4’s interaction with other proteins such as p53 [41,46].

**Figure 1:**
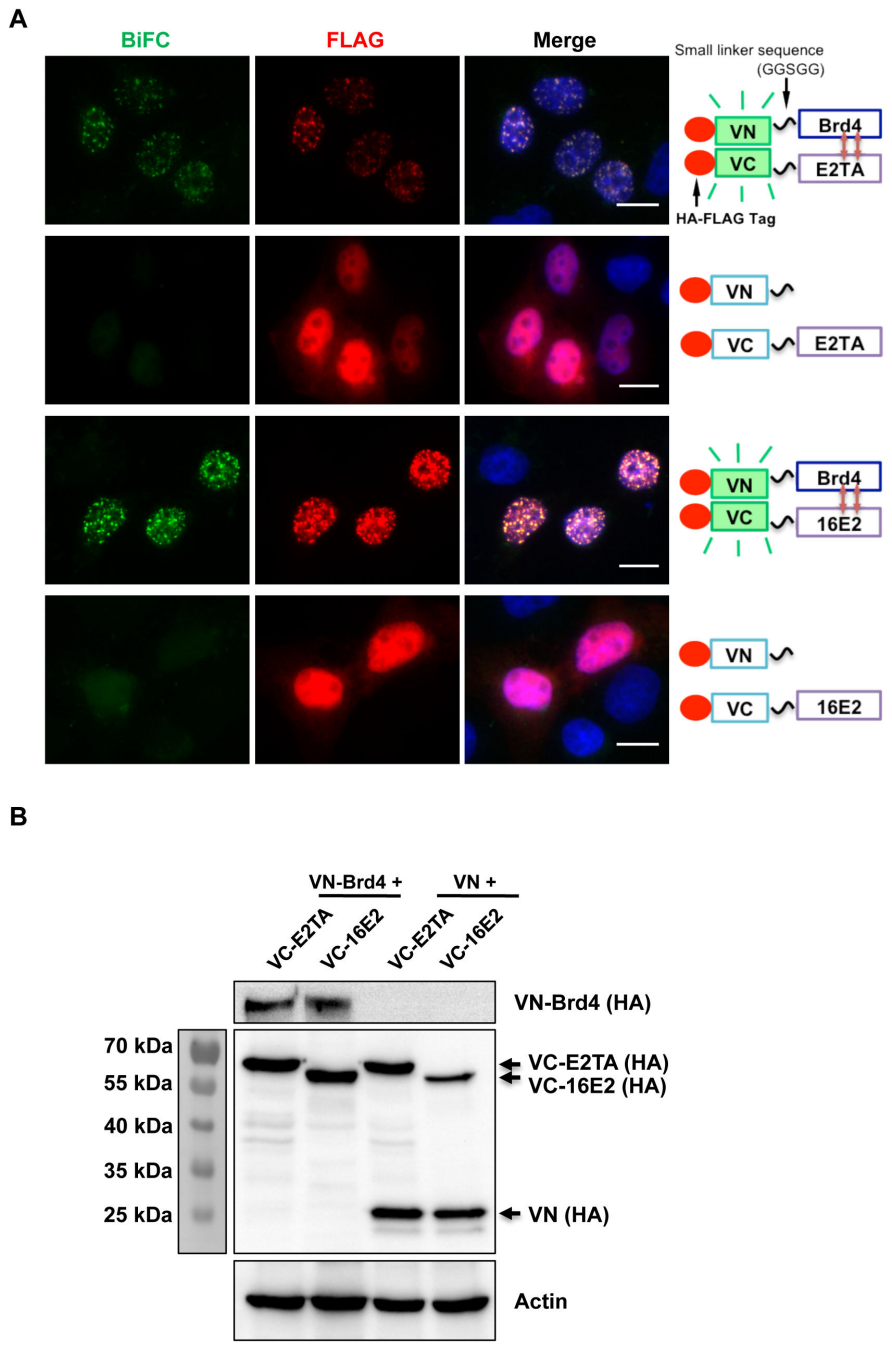
The BiFC signal is specifically generated through the E2-Brd4 interaction. (A) C33A cells were co-transfected with pairs of Venus N constructs (VN or VN-Brd4) and Venus C constructs (VC-E2TA or VC-16E2) as indicated on the right panel. Forty-eight hours post-transfection, the cells were fixed and stained with anti-FLAG antibody (red) and DAPI. Bar, 10 μm. (B) C33A cells were transfected as described in (A) and protein lysates were immunoblotted using ant-HA or anti-Actin antibodies. Arrows mark the VC-E2s or VN constructs expressed in cells.

Brd4 was fused to the N-terminal portion (VN) of the enhanced yellow fluorescent protein variant, Venus, while either HPV16 E2 or BPV1 E2TA was fused to the C-terminal portion of Venus (VC). Both of these constructs contain FLAG and hemagglutinin (HA) epitope tags to allow monitoring of the proteins in cells. In live C33A cells co-transfected with VN-Brd4 and either VC-E2TA or VC-16E2, we could detect punctate nuclear speckles of green BiFC signal, demonstrating a real-time interaction of Brd4 with BPV1 E2TA or HPV16 E2. These BiFC signals were reproducibly seen in fixed cells, so immunofluorescent staining was performed to allow high-resolution imaging. To confirm that the BiFC signal was specific for the E2-Brd4 interaction, the empty VN construct was co-transfected with either VC-E2TA or VC-16E2 into cells to determine if these pairs produce BiFC signal. As seen in Figure 1, while all the transfected constructs were expressed (as detected by anti-FLAG immuno-staining and immuno-blotting), only those cells co-expressing VN-Brd4 and a VC-tagged E2 protein generated BiFC signal. It is important to note that, in this experiment and all of the experiments described below, anti-FLAG immuno-staining was used to detect signals of both VN and VC proteins co-expressed in the cells whereas expression level of the individual proteins was determined using immuno-blotting. Although immuno-blotting shows low expression levels of VN-Brd4 and VC-tagged E2s, robust BiFC can be detected in cells co-expressing these proteins (Figure 1). On the other hand, even cells with a very strong FLAG signal of VN and a VC-E2 did not show BiFC signal (Figure 1A). In addition, co-expressing VN-Brd4 and VC proteins also did not generate BiFC signal ([41] and data not shown). These observations established that the BiFC was generated through specific interaction between E2 and Brd4.

**Figure 2:**
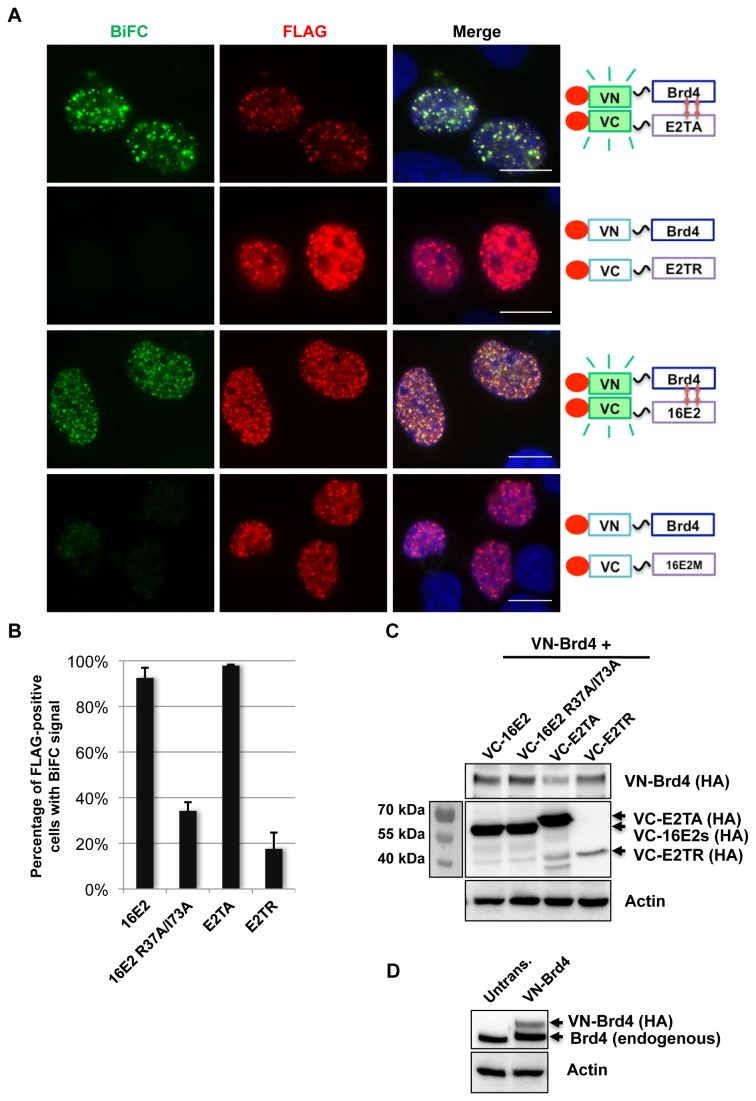
The E2-Brd4 BiFC signal is inhibited by mutating the Brd4 binding sites in E2. (A) C33A cells were co-transfected with VN-Brd4 and Venus C BiFC constructs (VC-E2TA, VC-E2TR, VC-16E2, or VC-16E2 R37A/I73A (16E2M)) as indicated on the right panel. Forty-eight hours post-transfection, the cells were fixed and stained with anti-FLAG antibody (red) and DAPI. Bar,10 μm. (B) For each transfection in (A), the percentage of cells showing BiFC signal was quantified from approximately 200 positively transfected cells. Average and standard deviation were calculated from three independent experiments. (C) C33A cells were either untreated or transfected as described in (A) and protein lysates were immunoblotted using anti-HA or anti-Actin antibodies. Arrows mark the VC-E2 constructs expressed in cells. (D) Protein lysates from untreated C33A cells or cells transfected with VN-Brd4 and VC-16E2 were immunoblotted using anti-Brd4 or anti-Actin antibodies.

We further tested the specificity of E2-Brd4 BiFC by examining E2 mutants defective in Brd4 binding. It has been previously shown that BPV1 E2TR does not bind Brd4 and that the HPV16 E2 R37A/I73A mutant protein binds Brd4 with much lower efficiency than its wild-type counterpart [20,31]. These E2 mutants were compared with their wild-type counterparts for interaction with Brd4 using BiFC (Figure 2). C33A cells were co-transfected with VN-Brd4 and one of the VC-E2s (E2TA, 16E2, E2TR, or 16E2 R37A/I73A). Expression level of the E2 constructs was shown by Western blot to be comparable except E2TR, which consistently had lower expression than the other E2s (Figure 2C). The VC-16E2 R37A/I73A therefore provided a better negative control for the wild E2s in BiFC. Notably, the VN-Brd4 fusion protein was expressed at a level much lower than endogenous Brd4 (Figure 2D), suggesting that it is not likely to induce an over-expression artifact. Co-expression of the wild-type E2s with Brd4 generated a strong BiFC signal in nuclear speckles (Figure 2A). In contrast, the E2 mutants only showed very dim BiFC nuclear foci (Figure 2A). The cells were also immuno-stained with anti-FLAG antibody to visualize co-expression of both FLAG-tagged Venus fusion proteins. The percentage of FLAG-positive cells with BiFC signal was also quantified (Figure 2B). While virtually all E2TA- or 16E2-transfected cells had robust BiFC signal, E2TR- or 16E2 R37A/I73A-transfected cells were either negative for BiFC signal or had very dim BiFC nuclear speckles (Figure 2A, bottom panel), which were counted as positive BiFC in the quantification. It is of note that the dim BiFC signal of 16E2 R37A/I73A-Brd4 or E2TR-Brd4 could only be detected in cells showing very high expression of Venus fusion proteins (as indicated by the strong FLAG signal). The large quantities of Brd4 and E2 mutant Venus fusion proteins present together in cells could contribute to the relatively high background BiFC, which was also observed in previous studies [47,48]. This study demonstrates that breaking the E2-Brd4 interaction by mutagenesis could greatly reduce the E2-Brd4 BiFC signal, confirming that the BiFC signal was specifically generated through the E2-Brd4 interaction. 

### BiFC analysis of the HPV16 E2-Brd4 interaction during the cell cycle

**Figure 3:**
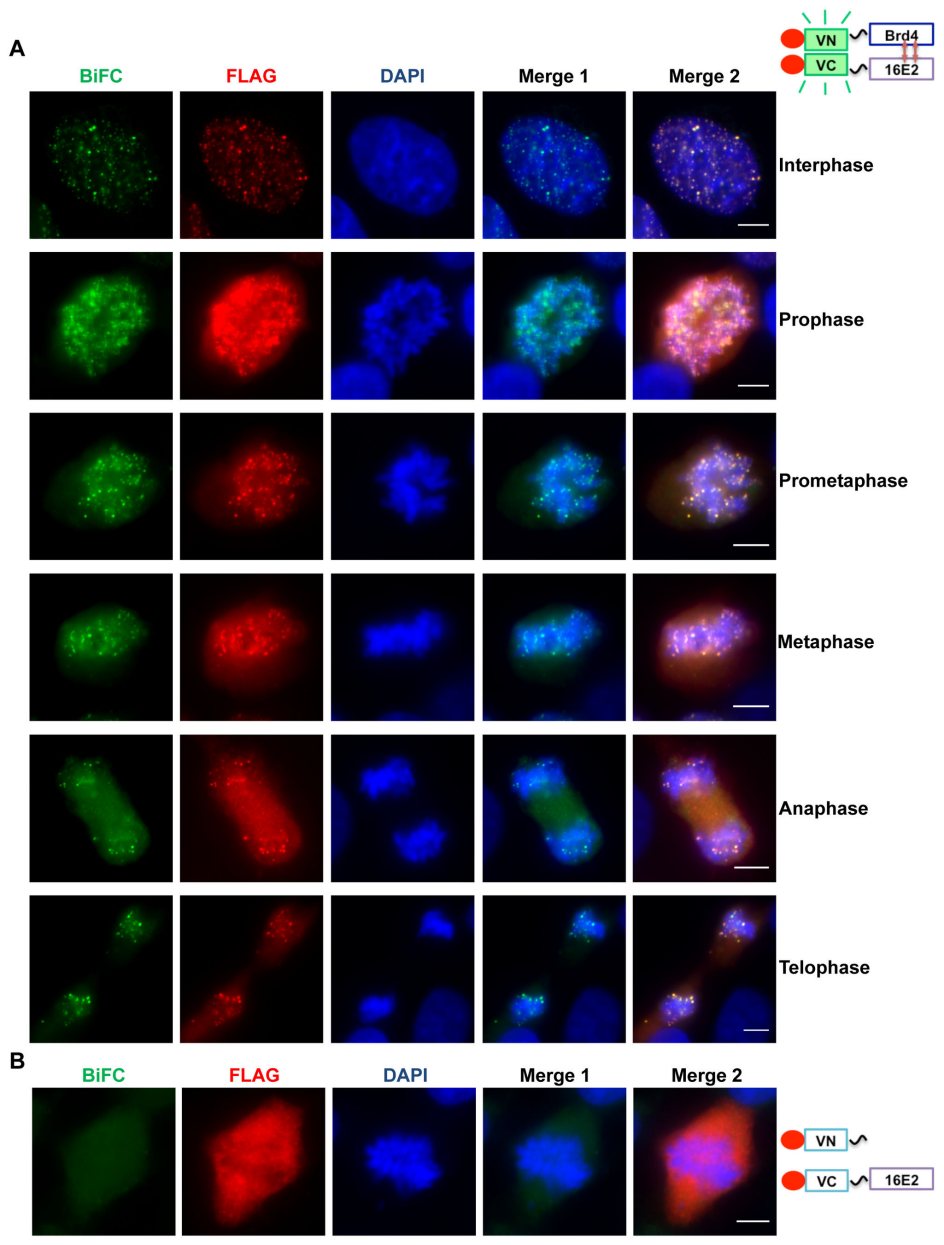
HPV16 E2 and Brd4 interact on chromatin in interphase and mitotic cells. (A) C33A cells were co-transfected with VN-Brd4 and VC-16E2 BiFC constructs. Forty-eight hours post-transfection, cells were fixed and stained with anti-FLAG antibody (red) and DAPI. Merge 1 is a merge of the BIFC and DAPI panels and Merge 2 is a merge of BIFC, FLAG and DAPI panels. (B) C33A cells were co-transfected with Venus N and VC-16E2 BiFC constructs. Forty-eight hours post-transfection, the cells were stained with anti-FLAG antibody (red) and DAPI. Bar, 5 μm.

Through immunofluorescence analyses, E2 proteins from various papillomavirus types have been shown to interact with Brd4 during interphase and mitosis [20]. However, this is less clear for the HPV16 E2-Brd4 interaction. In this study, we examined the interaction of HPV16 E2 with Brd4 during interphase and mitosis using the BiFC technique. C33A cells were co-transfected with VN-Brd4 and VC-16E2. In both live and fixed cells, we could detect 16E2-Brd4 BiFC fluorescence and FLAG staining in small dots on interphase chromatin as well as mitotic chromosomes from all phases of mitosis (Figure 3 and data not shown). Colocalization of 16E2 on mitotic chromosomes was Brd4-dependent because when VC-16E2 was co-expressed with an empty VN vector, no BiFC or FLAG signal was detected on mitotic chromosomes (Figure 3B). This experiment demonstrates that BiFC is a useful tool to study the E2-Brd4 interaction in live cells throughout the cell cycle. In addition, this study shows that HPV16 E2 and Brd4 interact on mitotic chromosomes throughout all stages of mitosis and suggests a potential role for Brd4 in the high-risk HPV E2-mediated episome tethering and maintenance in host cells.

### Disruption of the E2-Brd4 BiFC signal with the Brd4 CTD

**Figure 4:**
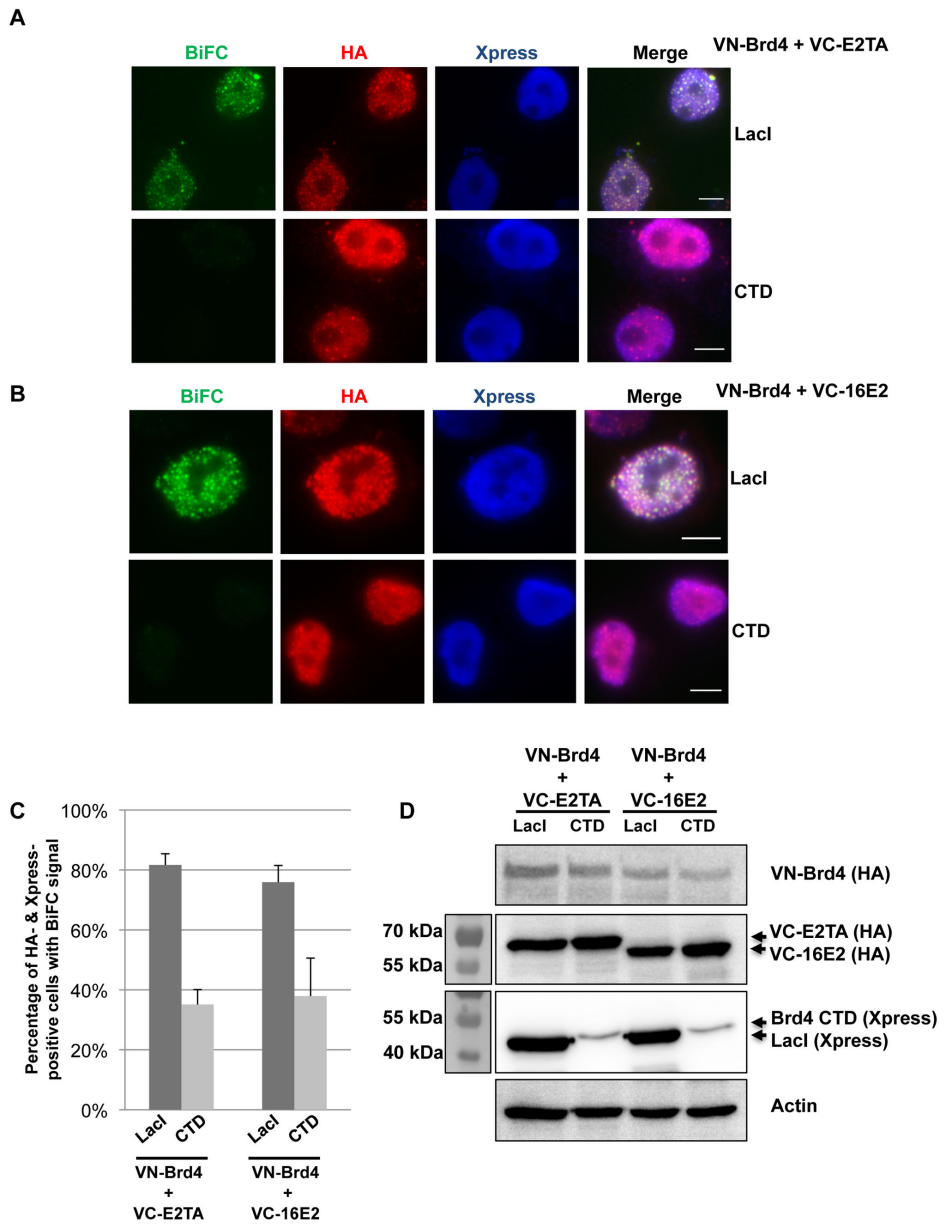
Brd4 CTD effectively disrupts the E2-Brd4 interaction measured by BiFC. (A, B) C33A cells were co-transfected with VN-Brd4, VC-E2TA or VC-16E2, and either Xpress-LacI or Xpress-Brd4 CTD constructs as indicated on the right panel. Forty-eight hours post-transfection, the cells were fixed and stained with anti-HA antibody (red) and anti-Xpress antibody (blue). (C) For each transfection in (A) and (B), the percentage of cells showing BiFC signal was quantified from approximately 100 Xpress- and HA-positive cells. Average and standard deviation were calculated from three independent experiments. Bar, 5 μm. (D) C33A cells were transfected as described in (A) and (B). Protein lysates were immunoblotted using anti-HA, anti-Xpress, or anti-Actin antibodies. Arrows mark the VC-E2s, Xpress-LacI, or Xpress-CTD constructs expressed in cells.

Brd4 binds PV E2 proteins through the CTD and we have previously demonstrated that the Brd4 CTD can competitively inhibit the E2-Brd4 interaction [31]. We wondered whether the Brd4 CTD could abrogate the E2-Brd4 BiFC signal. Therefore, C33A cells were co-transfected with VN-Brd4 plasmid, one of the VC-E2 constructs, and a plasmid expressing either Xpress-tagged Brd4 CTD or LacI, which is an irrelevant molecule that serves as a negative control (Figure 4). Both of these constructs contain nuclear localization signals to localize the proteins in the nucleus. Expression of each construct in this experiment was confirmed by Western blotting (Figure 4D). By examining the BiFC fluorescence of these samples in live cells, it was obvious that CTD transfection reduced the percentage of BiFC-positive cells as compared to LacI transfection (data not shown), even though the CTD was expressed at a much lower level than LacI (Figure 4D). To further confirm this result, we stained these cells with anti-HA antibody to detect the HA-tagged Venus fusion proteins, and with anti-Xpress antibody to detect Xpress-CTD or Xpress-LacI. As seen in Figures 4A and 4B, Xpress-LacI had no effect on the ability of VC-E2TA or VC-16E2 to interact with VN-Brd4 and to produce the BiFC signal. However, in Xpress-CTD-expressing cells, the E2-Brd4 BiFC fluorescence was markedly reduced. Quantification of the percentage of Xpress- and HA-positive cells with BiFC signal revealed a significant reduction in BiFC-positive cells when Xpress-CTD was expressed as compared to Xpress-LacI (Figure 4C). Even the cells with very dim BiFC signal were counted as positive; therefore the data in Figure 4C reflects a conservative quantification of the CTD effect. Nevertheless, this study showed that co-expression of a dominant negative inhibitor of the E2-Brd4 interaction could significantly reduce the BiFC signal, further confirming that the BiFC signal is generated through the specific E2-Brd4 interaction. This experiment also established BiFC as a useful method for studying the E2-Brd4 interaction in live cells and provided a proof of principle example for identifying inhibitors that could disrupt this interaction.

E2 is known to bind E2 binding sites on the viral genome as a dimer [49] and it has previously been shown that dimerized E2 interacts more efficiently with Brd4 [50]. We therefore predicted that the presence of HPV genome would promote E2 dimerization and enhance binding to Brd4. To test this hypothesis, we examined the HPV16 E2 and Brd4 BiFC signal in the presence of the HPV genome. The denaturing conditions of the immuno-FISH protocol dramatically quench BiFC fluorescence, precluding the use of FISH to visualize E2/Brd4 BiFC colocalization with HPV genomes. Therefore, we co-transfected C33A cells with VN-Brd4, VC-16E2, and either the HPV16 genome or an empty vector at a 1:2 ratio (BiFC constructs:HPV16 genome/vector) to increase the probability that cells with BiFC signal also contain HPV16 genomes or the control vector. Interestingly, cells co-transfected with HPV16 genome had a more than two-fold increase in E2-Brd4 BiFC signal intensity compared to vector co-transfected cells (Figure S1). These results suggest that E2 binding to the HPV genome enhances the E2-Brd4 interaction, likely because of efficient E2 dimerization. However, it is also possible that low-level expression of viral proteins from the HPV genome might somehow increase the E2-Brd4 interaction. Further experiments are needed to better understand this result. 

### E2-Brd4 BiFC is released from mitotic chromosomes after JQ1(+) treatment

**Figure 5:**
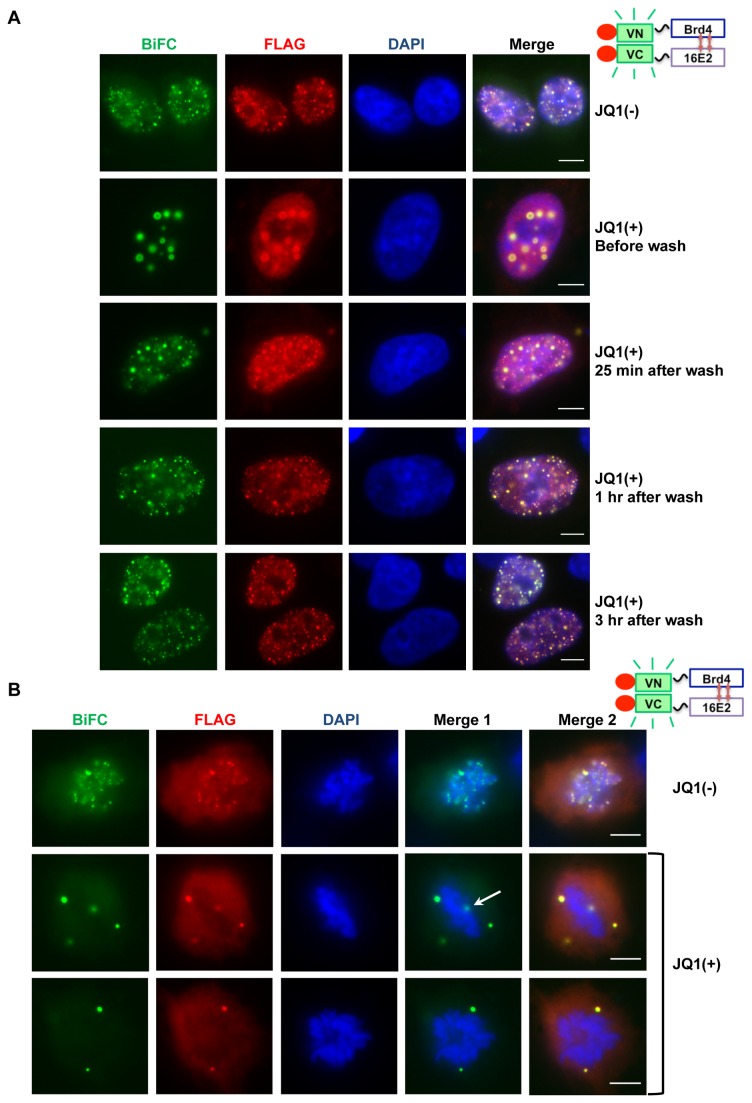
Releasing Brd4 from chromatin by JQ1(+) abolishes the E2-Brd4 interaction on mitotic chromosomes. (A) C33A cells were co-transfected with VN-Brd4 and VC-16E2 and treated with 500 nM JQ1(-) or JQ1(+) at 24h post transfection. Forty-eight hours post-transfection, cells were either fixed immediately (before wash) or washed several times and cultured for the times indicated on the right panel. All cells were fixed and stained with anti-FLAG antibody (red) and DAPI. (B) C33A cells were co-transfected with VN-Brd4 and VC-16E2. Forty-eight hours post-transfection, cells were treated with 500 nM JQ1(-) or JQ1(+) for 1h. The cells were then fixed and stained with anti-FLAG antibody (red) and DAPI. Merge 1 is a merge of the BIFC and DAPI panels and Merge 2 is a merge of BIFC, FLAG and DAPI panels. Bar, 5 μm. The white arrow marks a BiFC focus superimposed on a mitotic chromosome.

The chemical compound JQ1(+) binds to and inhibits Brd4 bromodomain binding to acetylated histones, leading to effective release of Brd4 from chromatin [44]. Since Brd4 is thought to tether E2 to mitotic chromosomes for viral genome maintenance, we tested if releasing Brd4 from chromosomes by JQ1(+) disrupts the association of the E2-Brd4 complex with mitotic chromosomes. We therefore used the BiFC system to test the effect of JQ1(+) on the E2-Brd4 interaction with interphase chromatin and mitotic chromosomes. C33A cells were co-transfected with VN-Brd4 and VC-16E2 constructs and, 24 hours later, treated with 500 nM JQ1(+) or the inactive enantiomer JQ1(-) for various amounts of time. Chromatin immunoprecipitation analysis confirmed that 500 nM JQ1(+) could efficiently dissociate Brd4 from chromatin while 500 nM JQ1(-) had no detectable effect (data not shown). After only 30 minutes of JQ1(+) treatment, E2-Brd4 BiFC was no longer bound to chromatin in small speckles but formed punctate foci, which grew larger as the incubation time with JQ1(+) increased (Figure 5A and data not shown). In contrast, cells treated with JQ1(-) showed normal E2-Brd4 BiFC speckles on chromatin throughout the analysis (Figure 5A). Figure 5A shows the BiFC patterns in the large majority of cells treated under different conditions. 

To rule out the possibility that these punctate foci were non-specific aggregates of the BiFC proteins, we co-transfected the empty VC construct with VN-Brd4 into cells to determine if these pairs produce large BiFC foci after JQ1(+) treatment. As in Figure 1, no BiFC signal was detected in these cells. In contrast to the enrichment of FLAG immuno-staining signal in large foci in JQ1(+) treated E2/Brd4-expressing cells (Figure 5A), the VN-Brd4 FLAG immunofluorescent signal was not localized into foci (data not shown). This result suggests that the large foci we detected with JQ1(+) in cells co-transfected with VN-Brd4 and VC-16E2 constructs are specific for the E2/Brd4 BiFC pair and are not likely to be non-specific protein aggregates.

To further analyze the specificity of these foci, we tested whether the E2-Brd4 BiFC in punctate foci would return to normal speckles on chromatin when JQ1(+) was washed away. Cells transfected with the E2/Brd4 BiFC constructs were cultured with JQ1(+) for 24 hours and then thoroughly washed and cultured for up to three more hours without JQ1(+). Remarkably, the E2-Brd4 BiFC began returning to its usual speckled pattern on chromatin only five minutes after removing JQ1(+) and, in most cells, was completely restored to its normal cellular localization pattern after one hour (Figure 5A and data not shown). Similar results were also observed with VN-Brd4 and VC-E2TA (data not shown). This additional evidence suggests that these foci are not likely to be non-specific protein aggregates but rather large, reversible E2-Brd4 complexes formed when Brd4 is released from chromatin.

 We next analyzed the effect of JQ1(+) on the 16E2-Brd4 association with mitotic chromosomes. C33A cells were transfected with the 16E2/Brd4 BiFC constructs and, 48 hours later, treated with 500 nM JQ1(+) or JQ1(-) for one hour. This short JQ1 treatment was intended to minimize its effects on the cell cycle and BiFC protein levels. Similar to the observation made without drug treatment (Figure 3), cells treated with JQ1(-) showed the 16E2-Brd4 BiFC signal and FLAG staining in small speckles associated with chromosomes in 36 out of 51 (70.6%) transfected mitotic cells (Figure 5B). Conversely, the 16E2-Brd4 BiFC in mitotic cells treated with JQ1(+) was in large foci clearly dissociated from chromosomes and only 6 out of 50 (16.0%) transfected mitotic cells had E2-Brd4 BiFC that appeared on chromosomes (Figure 5B). These foci were likely already dissociated from the chromosomes while their signals superimposed on the chromosomes under the microscope (Figure 5B, white arrow). Since it was impossible to determine for sure, we counted these cells as having E2-Brd4 BiFC still associated with chromatin, making the above quantification a conservative estimate. Notably, in JQ1(+) treated cells, a much smaller number of E2-Brd4 BiFC foci was detected compared to the JQ1(-) treated cells, indicating that, when excluded from chromosomes during mitosis, the interaction between these two proteins may become less stable. It is important to note that FLAG staining signal was also excluded from mitotic chromosomes in JQ1(+) treated cells (Figure 5B), suggesting that 16E2 binding to mitotic chromosomes is dependent on Brd4’s association with chromatin. Similar JQ1(+) effect on the E2TA-Brd4 association with mitotic chromosomes was observed (data not shown). These results reveal the potential of JQ1(+) as a possible antiviral tool for disruption of HPV episome maintenance during persistent infection and suggest an important role of Brd4 for tethering HPV16 E2 to mitotic chromosomes.

## Discussion

The HPV vaccines are invaluable as preventative treatment against HPV infection and in the long term will likely reduce the worldwide prevalence of infection by the HPV subtypes 6, 11, 16, and 18. However, there is still a great need for antiviral drugs to treat current HPV infections of a variety of other HPV subtypes. The E2-Brd4 interaction is an attractive drug target because this complex mediates multiple functions in the HPV life cycle, including viral transcription, genome replication, and episome maintenance. Indeed, it has been previously shown that abolishing HPV16 E2’s association with Brd4 using E2 mutants or Brd4 CTD impairs viral replication, inhibits gene transcription, and releases HPV genomes from mitotic chromosomes, suggesting that breaking this interaction could disrupt multiple stages of the HPV life cycle [20,21,36,39,51]. 

In this study, we used BiFC technology to visualize the E2-Brd4 interaction in both live and fixed cells. We detected strong BiFC nuclear signal in the majority of cells co-expressing VN-Brd4 and either VC-16E2 or VC-E2TA. These nuclear speckles resemble the punctate immunofluorescence colocalization pattern seen previously for E2 and Brd4 [31]. The E2-Brd4 BiFC signal was significantly abolished by mutating the Brd4 binding sites in E2 or by a dominant negative inhibitor of the E2-Brd4 interaction, suggesting that this signal is generated through the specific interaction between E2 and Brd4. Additionally, the 16E2-Brd4 interaction was detected on both interphase chromatin and mitotic chromosomes in all phases of mitosis. This study demonstrates that the BiFC technique is a useful tool to study the E2-Brd4 interaction in live cells throughout the cell cycle, establishing its potential for examining this virus and host protein interaction during the differentiation-dependent viral life cycle. This technique could also be used for studying when, during the HPV life cycle, E2 binds Brd4 for tethering and then E1 for replication. 

Brd4 normally associates with acetylated histones with a rapid “on and off” dynamic, and E2 binding Brd4 has been shown to stabilize Brd4’s chromatin association [32,34]. In addition, Brd4 is likely a mitotic chromosome tether for HPV episome maintenance because inhibiting the E2-Brd4 interaction results in episome dissociation from chromosomes [36]. However, whether HPV16 associates with mitotic chromosomes through Brd4 has not been clearly resolved. Therefore the association of the 16E2-Brd4 complex with mitotic chromosomes during all stages of mitosis observed in this study represents a novel finding for the HPV16 E2. This result indicates that HPV16 E2, like BPV E2TA, may also associate with Brd4 for tethering viral genomes to mitotic chromosomes to ensure faithful partitioning of the viral genomes to daughter cells during mitosis.

We also used the BiFC system to determine if small molecule JQ1(+)-mediated dissociation of Brd4 from chromatin affects 16E2-Brd4 binding to mitotic chromosomes. Interestingly, when released from chromatin by JQ1(+), the E2-Brd4 BiFC formed multiple, punctate nuclear spheres, which grew larger with increased JQ1(+) incubation time. These large foci were not detected in cells treated with JQ1(-) nor in cells co-transfected with VN-Brd4 and the empty VC plasmid, indicating that they were specifically formed by the E2-Brd4 BiFC proteins when Brd4 was released from histones. This foci formation process was reversible as the E2-Brd4 BiFC foci rapidly returned to chromatin-associated speckles in most cells when JQ1(+) was removed. Furthermore, Brd4 dissociation from chromatin dramatically reduced the number of mitotic cells with chromosome-associated E2-Brd4 BiFC, pointing to the possibility that JQ1(+) can be used as a potential drug to disrupt HPV episome maintenance and to clear HPV persistent infections. 

In a recent study from our laboratory, we found that Brd4 is recruited to HPV replication foci and is essential for viral replication [39]. We further demonstrated that Brd4 dissociation from chromatin by JQ1(+) stimulates HPV genome replication and postulated that Brd4 released from chromatin might be recruited to the HPV replication complex to support viral replication. In line with this model, the current study shows that E2-Brd4 BiFC signal localizes to nuclear foci when cells were treated with JQ1(+). Since HPV genome amplification is normally restricted to terminally differentiated cells to evade host immune surveillance, the JQ1(+)-induced impromptu viral genome amplification in the infected basal cells could trigger activation of the immune response. From these findings, we hypothesize that JQ1(+)’s combined effects on abolishing viral episome maintenance and stimulating premature viral genome amplification in latently infected cells could result in activation of the immune response and clearance of viral genomes before infected cells could develop into cancer. Obviously, to test this hypothesis, further studies are needed to investigate the overall impact of JQ1(+) on the HPV life cycle. 

In summary, this study used the BiFC technique to recapitulate the PV E2 interaction with the host receptor Brd4 in live cells throughout all stages of the host cell cycle. The E2-Brd4 BiFC assay established in live cells provides a useful platform for screening small molecule inhibitors of this important virus-host interaction as anti-HPV drugs. This study also demonstrates general implication for using BiFC as an effective tool for identifying inhibitors of important protein-protein interactions. This will hopefully facilitate the screening of antiviral drugs, as well as inhibitors of key interactions for various cancers and other diseases. The JQ1(+)-mediated dissociation of Brd4 from chromatin also provides support for using this small molecule to disrupt HPV episome maintenance and to treat HPV persistent infection. 

## Supporting Information

Figure S1 **The E2-Brd4 BiFC signal is enhanced by the presence of HPV16 genome.** (A) C33A cells were co-transfected with VN-Brd4, VC-16E2, and either pUC19 or pEFHPV-16W12E at a 1:2 ratio. Forty-eight hours post-transfection, cells were fixed and stained with anti-FLAG antibody (red) and DAPI. Bar, 5 μm. In the vector control, the BiFC signal is dimmer than in previous figures because there is much less E2/Brd4 BiFC DNA transfected. (B) Scatter plot of the average BiFC signal intensity divided by nucleus area in cells transfected as in (A). Data were collected from 50 vector transfected cells and 50 HPV16 genome transfected cells using ImageJ. This experiment was repeated twice with similar results. Bars indicate the mean of all cells examined.
(TIF)

## References

[1] zur Hausen H (2009) Papillomaviruses in the causation of human cancers - a brief historical account. Virology 384: 260-265. doi:10.1016/j.virol.2008.11.046. PubMed: 19135222. 19135222

[2] Muñoz N, Bosch FX, de Sanjosé S, Herrero R, Castellsagué X et al. (2003) Epidemiologic classification of human papillomavirus types associated with cervical cancer. N Engl J Med 348: 518-527. doi:10.1056/NEJMoa021641. PubMed: 12571259. 12571259

[3] Smith JS, Lindsay L, Hoots B, Keys J, Franceschi S et al. (2007) Human papillomavirus type distribution in invasive cervical cancer and high-grade cervical lesions: a meta-analysis update. Int J Cancer 121: 621-632. doi:10.1002/ijc.22527. PubMed: 17405118. 17405118

[4] Walboomers JM, Jacobs MV, Manos MM, Bosch FX, Kummer JA et al. (1999) Human papillomavirus is a necessary cause of invasive cervical cancer worldwide. J Pathol 189: 12-19. doi:10.1002/(SICI)1096-9896(199909)189:1. PubMed: 10451482. 10451482

[5] Doorbar J (2006) Molecular biology of human papillomavirus infection and cervical cancer. Clin Sci (Lond) 110: 525-541. doi:10.1042/CS20050369. PubMed: 16597322. 16597322

[6] Kjaer SK, Sigurdsson K, Iversen OE, Hernandez-Avila M, Wheeler CM et al. (2009) A pooled analysis of continued prophylactic efficacy of quadrivalent human papillomavirus (Types 6/11/16/18) vaccine against high-grade cervical and external genital lesions. Cancer. Prev Res (Phila) 2: 868-878. doi:10.1158/1940-6207.CAPR-09-0031. 19789295

[7] Paavonen J, Naud P, Salmerón J, Wheeler CM, Chow SN et al. (2009) Efficacy of human papillomavirus (HPV)-16/18 AS04-adjuvanted vaccine against cervical infection and precancer caused by oncogenic HPV types (PATRICIA): final analysis of a double-blind, randomised study in young women. Lancet 374: 301-314. doi:10.1016/S0140-6736(09)61248-4. PubMed: 19586656. 19586656

[8] Dillner J, Kjaer SK, Wheeler CM, Sigurdsson K, Iversen OE et al. (2010) Four year efficacy of prophylactic human papillomavirus quadrivalent vaccine against low grade cervical, vulvar, and vaginal intraepithelial neoplasia and anogenital warts: randomised controlled trial. BMJ 341: c3493. doi:10.1136/bmj.c3493. PubMed: 20647284. 20647284 PMC2907480

[9] Trimble CL, Peng S, Kos F, Gravitt P, Viscidi R et al. (2009) A phase I trial of a human papillomavirus DNA vaccine for HPV16+ cervical intraepithelial neoplasia 2/3. Clin Cancer Res 15: 361-367. doi:10.1158/1078-0432.CCR-08-1725. PubMed: 19118066. 19118066 PMC2865676

[10] Ault KA (2007) Human papillomavirus vaccines and the potential for cross-protection between related HPV types. Gynecol Oncol 107: S31-S33. doi:10.1016/j.ygyno.2007.08.059. PubMed: 18499916. 18499916

[11] Stanley MA (2012) Genital human papillomavirus infections: current and prospective therapies. J Gen Virol 93: 681-691. doi:10.1099/vir.0.039677-0. PubMed: 22323530. 22323530

[12] White PW, Faucher AM, Massariol MJ, Welchner E, Rancourt J et al. (2005) Biphenylsulfonacetic acid inhibitors of the human papillomavirus type 6 E1 helicase inhibit ATP hydrolysis by an allosteric mechanism involving tyrosine 486. Antimicrob Agents Chemother 49: 4834-4842. doi:10.1128/AAC.49.12.4834-4842.2005. PubMed: 16304143. 16304143 PMC1315966

[13] White PW, Faucher AM, Goudreau N (2011) Small molecule inhibitors of the human papillomavirus E1-E2 interaction. Curr Top Microbiol Immunol 348: 61-88. PubMed: 20676971. 20676971 10.1007/82_2010_92

[14] McBride AA, Romanczuk H, Howley PM (1991) The papillomavirus E2 regulatory proteins. J Biol Chem 266: 18411-18414. PubMed: 1655748. 1655748

[15] Spalholz BA, Yang YC, Howley PM (1985) Transactivation of a bovine papilloma virus transcriptional regulatory element by the E2 gene product. Cell 42: 183-191. doi:10.1016/S0092-8674(85)80114-8. PubMed: 2990724. 2990724

[16] Kim J, Lee D, Gwan Hwang S, Hwang ES, Choe J (2003) BRCA1 associates with human papillomavirus type 18 E2 and stimulates E2-dependent transcription. Biochem Biophys Res Commun 305: 1008-1016. doi:10.1016/S0006-291X(03)00880-5. PubMed: 12767931. 12767931

[17] Kumar RA, Naidu SR, Wang X, Imbalzano AN, Androphy EJ (2007) Interaction of papillomavirus E2 protein with the Brm chromatin remodeling complex leads to enhanced transcriptional activation. J Virol 81: 2213-2220. doi:10.1128/JVI.01746-06. PubMed: 17151122. 17151122 PMC1865958

[18] Lee D, Hwang SG, Kim J, Choe J (2002) Functional interaction between p/CAF and human papillomavirus E2 protein. J Biol Chem 277: 6483-6489. doi:10.1074/jbc.M105085200. PubMed: 11744716. 11744716

[19] Lee D, Lee B, Kim J, Kim DW, Choe J (2000) cAMP response element-binding protein-binding protein binds to human papillomavirus E2 protein and activates E2-dependent transcription. J Biol Chem 275: 7045-7051. doi:10.1074/jbc.275.10.7045. PubMed: 10702269. 10702269

[20] McPhillips MG, Oliveira JG, Spindler JE, Mitra R, McBride AA (2006) Brd4 is required for e2-mediated transcriptional activation but not genome partitioning of all papillomaviruses. J Virol 80: 9530-9543. doi:10.1128/JVI.01105-06. PubMed: 16973557. 16973557 PMC1617221

[21] Schweiger MR, You J, Howley PM (2006) Bromodomain protein 4 mediates the papillomavirus E2 transcriptional activation function. J Virol 80: 4276-4285. doi:10.1128/JVI.80.9.4276-4285.2006. PubMed: 16611886. 16611886 PMC1472042

[22] Schweiger MR, Ottinger M, You J, Howley PM (2007) Brd4-independent transcriptional repression function of the papillomavirus e2 proteins. J Virol 81: 9612-9622. doi:10.1128/JVI.00447-07. PubMed: 17626100. 17626100 PMC2045424

[23] Smith JA, White EA, Sowa ME, Powell ML, Ottinger M et al. (2010) Genome-wide siRNA screen identifies SMCX, EP400, and Brd4 as E2-dependent regulators of human papillomavirus oncogene expression. Proc Natl Acad Sci U S A 107: 3752-3757. doi:10.1073/pnas.0914818107. PubMed: 20133580. 20133580 PMC2840515

[24] Tan SH, Leong LE, Walker PA, Bernard HU (1994) The human papillomavirus type 16 E2 transcription factor binds with low cooperativity to two flanking sites and represses the E6 promoter through displacement of Sp1 and TFIID. J Virol 68: 6411-6420. PubMed: 8083979. 8083979 10.1128/jvi.68.10.6411-6420.1994 PMC237061

[25] Wu SY, Lee AY, Hou SY, Kemper JK, Erdjument-Bromage H et al. (2006) Brd4 links chromatin targeting to HPV transcriptional silencing. Genes Dev 20: 2383-2396. doi:10.1101/gad.1448206. PubMed: 16921027. 16921027 PMC1560413

[26] Skiadopoulos MH, McBride AA (1998) Bovine papillomavirus type 1 genomes and the E2 transactivator protein are closely associated with mitotic chromatin. J Virol 72: 2079-2088. PubMed: 9499063. 9499063 10.1128/jvi.72.3.2079-2088.1998 PMC109502

[27] Bastien N, McBride AA (2000) Interaction of the papillomavirus E2 protein with mitotic chromosomes. Virology 270: 124-134. doi:10.1006/viro.2000.0265. PubMed: 10772985. 10772985

[28] Piirsoo M, Ustav E, Mandel T, Stenlund A, Ustav M (1996) Cis and trans requirements for stable episomal maintenance of the BPV-1 replicator. EMBO J 15: 1-11. PubMed: 8598191. 8598191 PMC449912

[29] Ilves I, Kivi S, Ustav M (1999) Long-term episomal maintenance of bovine papillomavirus type 1 plasmids is determined by attachment to host chromosomes, which Is mediated by the viral E2 protein and its binding sites. J Virol 73: 4404-4412. PubMed: 10196338. 10196338 10.1128/jvi.73.5.4404-4412.1999 PMC104221

[30] Lehman CW, Botchan MR (1998) Segregation of viral plasmids depends on tethering to chromosomes and is regulated by phosphorylation. Proc Natl Acad Sci U S A 95: 4338-4343. doi:10.1073/pnas.95.8.4338. PubMed: 9539738. 9539738 PMC22490

[31] You J, Croyle JL, Nishimura A, Ozato K, Howley PM (2004) Interaction of the bovine papillomavirus E2 protein with Brd4 tethers the viral DNA to host mitotic chromosomes. Cell 117: 349-360. doi:10.1016/S0092-8674(04)00402-7. PubMed: 15109495. 15109495

[32] Dey A, Chitsaz F, Abbasi A, Misteli T, Ozato K (2003) The double bromodomain protein Brd4 binds to acetylated chromatin during interphase and mitosis. Proc Natl Acad Sci U S A 100: 8758-8763. doi:10.1073/pnas.1433065100. PubMed: 12840145. 12840145 PMC166386

[33] Baxter MK, McPhillips MG, Ozato K, McBride AA (2005) The mitotic chromosome binding activity of the papillomavirus E2 protein correlates with interaction with the cellular chromosomal protein, Brd4. J Virol 79: 4806-4818. doi:10.1128/JVI.79.8.4806-4818.2005. PubMed: 15795266. 15795266 PMC1069523

[34] McPhillips MG, Ozato K, McBride AA (2005) Interaction of bovine papillomavirus E2 protein with Brd4 stabilizes its association with chromatin. J Virol 79: 8920-8932. doi:10.1128/JVI.79.14.8920-8932.2005. PubMed: 15994786. 15994786 PMC1168793

[35] Brannon AR, Maresca JA, Boeke JD, Basrai MA, McBride AA (2005) Reconstitution of papillomavirus E2-mediated plasmid maintenance in Saccharomyces cerevisiae by the Brd4 bromodomain protein. Proc Natl Acad Sci U S A 102: 2998-3003. doi:10.1073/pnas.0407818102. PubMed: 15710895. 15710895 PMC549465

[36] Abbate EA, Voitenleitner C, Botchan MR (2006) Structure of the papillomavirus DNA-tethering complex E2:Brd4 and a peptide that ablates HPV chromosomal association. Mol Cell 24: 877-889. doi:10.1016/j.molcel.2006.11.002. PubMed: 17189190. 17189190

[37] Ilves I, Mäemets K, Silla T, Janikson K, Ustav M (2006) Brd4 is involved in multiple processes of the bovine papillomavirus type 1 life cycle. J Virol 80: 3660-3665. doi:10.1128/JVI.80.7.3660-3665.2006. PubMed: 16537635. 16537635 PMC1440376

[38] Gagnon D, Joubert S, Sénéchal H, Fradet-Turcotte A, Torre S et al. (2009) Proteasomal degradation of the papillomavirus E2 protein is inhibited by overexpression of bromodomain-containing protein 4. J Virol 83: 4127-4139. doi:10.1128/JVI.02468-08. PubMed: 19211738. 19211738 PMC2668459

[39] Wang X, Helfer CM, Pancholi N, Bradner JE, You J (2013) Recruitment of Brd4 to the human papillomavirus type 16 DNA replication complex is essential for replication of viral DNA. J Virol 87: 3871-3884. doi:10.1128/JVI.03068-12. PubMed: 23365439. 23365439 PMC3624215

[40] Yan J, Li Q, Lievens S, Tavernier J, You J (2010) Abrogation of the Brd4-positive transcription elongation factor B complex by papillomavirus E2 protein contributes to viral oncogene repression. J Virol 84: 76-87. doi:10.1128/JVI.01647-09. PubMed: 19846528. 19846528 PMC2798453

[41] Wang R, Li Q, Helfer CM, Jiao J, You J (2012) The bromodomain protein Brd4 associated with acetylated chromatin is important for maintenance of higher-order chromatin structure. J Biol Chem. 10.1074/jbc.M111.323493 PMC3322821 22334664

[42] Flores ER, Allen-Hoffmann BL, Lee D, Sattler CA, Lambert PF (1999) Establishment of the human papillomavirus type 16 (HPV-16) life cycle in an immortalized human foreskin keratinocyte cell line. Virology 262: 344-354. doi:10.1006/viro.1999.9868. PubMed: 10502513. 10502513

[43] Yan J, Diaz J, Jiao J, Wang R, You J (2011) Perturbation of BRD4 protein function by BRD4-NUT protein abrogates cellular differentiation in NUT midline carcinoma. J Biol Chem 286: 27663-27675. doi:10.1074/jbc.M111.246975. PubMed: 21652721. 21652721 PMC3149357

[44] Filippakopoulos P, Qi J, Picaud S, Shen Y, Smith WB et al. (2010) Selective inhibition of BET bromodomains. Nature 468: 1067-1073. doi:10.1038/nature09504. PubMed: 20871596. 20871596 PMC3010259

[45] Kerppola TK (2006) Design and implementation of bimolecular fluorescence complementation (BiFC) assays for the visualization of protein interactions in living cells. Nat Protoc 1: 1278-1286. doi:10.1038/nprot.2006.201. PubMed: 17406412. 17406412 PMC2518326

[46] Wu SY, Lee AY, Lai HT, Zhang H, Chiang CM (2013) Phospho switch triggers Brd4 chromatin binding and activator recruitment for gene-specific targeting. Mol Cell 49: 843-857. doi:10.1016/j.molcel.2012.12.006. PubMed: 23317504. 23317504 PMC3595396

[47] Kodama Y, Hu CD (2010) An improved bimolecular fluorescence complementation assay with a high signal-to-noise ratio. BioTechniques 49: 793-805. doi:10.2144/000113519. PubMed: 21091444. 21091444

[48] Nakagawa C, Inahata K, Nishimura S, Sugimoto K (2011) Improvement of a Venus-based bimolecular fluorescence complementation assay to visualize bFos-bJun interaction in living cells. Biosci Biotechnol Biochem 75: 1399-1401. doi:10.1271/bbb.110189. PubMed: 21737916. 21737916

[49] Dostatni N, Thierry F, Yaniv M (1988) A dimer of BPV-1 E2 containing a protease resistant core interacts with its DNA target. EMBO J 7: 3807-3816. PubMed: 2850174. 2850174 10.1002/j.1460-2075.1988.tb03265.x PMC454957

[50] Cardenas-Mora J, Spindler JE, Jang MK, McBride AA (2008) Dimerization of the papillomavirus E2 protein is required for efficient mitotic chromosome association and Brd4 binding. J Virol 82: 7298-7305. doi:10.1128/JVI.00772-08. PubMed: 18495759. 18495759 PMC2493320

[51] Sénéchal H, Poirier GG, Coulombe B, Laimins LA, Archambault J (2007) Amino acid substitutions that specifically impair the transcriptional activity of papillomavirus E2 affect binding to the long isoform of Brd4. Virology 358: 10-17. doi:10.1016/j.virol.2006.08.035. PubMed: 17023018. 17023018

