# Supplementary figures and images for: Analysis of the Papillomavirus E2 and Bromodomain Protein Brd4 Interaction Using Bimolecular Fluorescence Complementation
Source: PLoS One. 2013 Oct 25;8(10):e77994. doi: 10.1371/journal.pone.0077994 (PMC3808292; doi:10.1371/journal.pone.0077994)

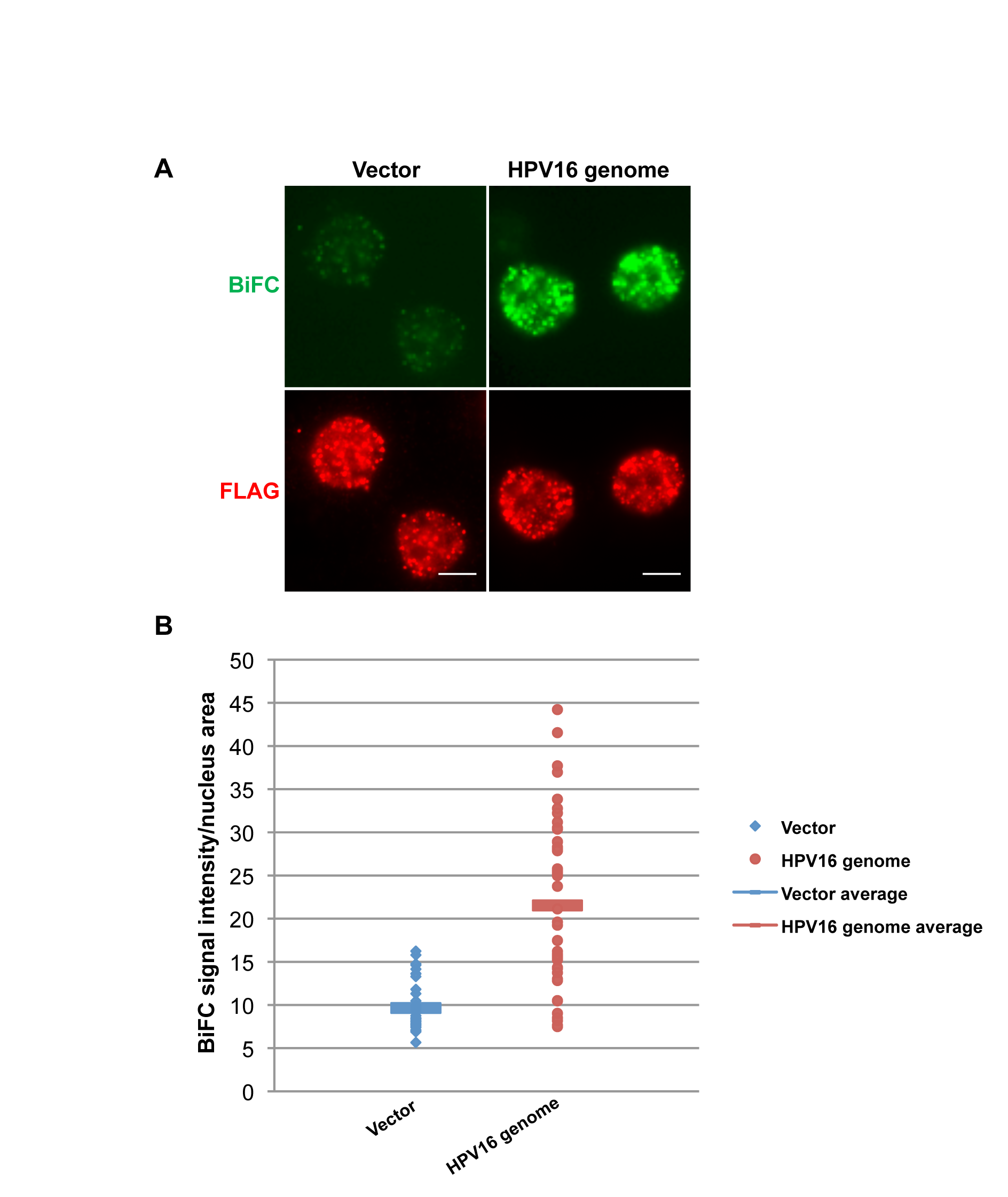

Supplement: Figure S1 — The E2-Brd4 BiFC signal is enhanced by the presence of HPV16 genome. (A) C33A cells were co-transfected with VN-Brd4, VC-16E2, and either pUC19 or pEFHPV-16W12E at a 1:2 ratio. Forty-eight hours post-transfection, cells were fixed and stained with anti-FLAG antibody (red) and DAPI. Bar, 5 μm. In the vector control, the BiFC signal is dimmer than in previous figures because there is much less E2/Brd4 BiFC DNA transfected. (B) Scatter plot of the average BiFC signal intensity divided by nucleus area in cells transfected as in (A). Data were collected from 50 vector transfected cells and 50 HPV16 genome transfected cells using ImageJ. This experiment was repeated twice with similar results. Bars indicate the mean of all cells examined. (TIF) [file pone.0077994.s001.tif]
